# Multiplicative modulations enhance diversity of hue-selective cells

**DOI:** 10.1038/s41598-020-64969-3

**Published:** 2020-05-22

**Authors:** Paria Mehrani, Andrei Mouraviev, John K. Tsotsos

**Affiliations:** The Center for Vision Research, York University, Toronto, M3J 1P3 Canada

**Keywords:** Colour vision, Network models

## Abstract

There is still much to understand about the brain’s colour processing mechanisms and the transformation from cone-opponent representations to perceptual hues. Moreover, it is unclear which area(s) in the brain represent unique hues. We propose a hierarchical model inspired by the neuronal mechanisms in the brain for local hue representation, which reveals the contributions of each visual cortical area in hue representation. Hue encoding is achieved through incrementally increasing processing nonlinearities beginning with cone input. Besides employing nonlinear rectifications, we propose multiplicative modulations as a form of nonlinearity. Our simulation results indicate that multiplicative modulations have significant contributions in encoding of hues along intermediate directions in the MacLeod-Boynton diagram and that our model V2 neurons have the capacity to encode unique hues. Additionally, responses of our model neurons resemble those of biological colour cells, suggesting that our model provides a novel formulation of the brain’s colour processing pathway.

## Introduction

The colour processing mechanisms in the primary visual cortex and later processing stages are a target of debate among colour vision researchers. What is also unclear is which brain area represents unique hues, those pure colours unmixed with other colours. In spite of a lack of agreement on colour mechanisms in higher visual areas, human visual system studies confirm that colour encoding starts with three types of cones sensitive to long (L), medium (M) and short (S) wavelengths, forming the LMS colour space. Cones send opponent feedforward signals to LGN cells with single-opponent receptive fields^1–3^. Cone-opponent mechanisms such as those in LGN were the basis of the “Opponent Process Theory” of Hering^4^, where he introduced unique hues. The four unique hues, red, green, yellow and blue were believed to be encoded by cone-opponent processes. Later studies^5–7^, however, confirmed that the cone-opponent mechanisms of earlier processing stages do not correspond to Hering’s red vs. green and yellow vs. blue opponent processes. In fact, they observed that the colour coding in early stages is organized along the two dimensions of the MacLeod and Boynton (MB)^8^ diagram, where abscissa and ordinate represent L vs. M and S vs. LM activations respectively.

Beyond LGN, studies on multiple regions in the ventral stream show an increase in nonlinearity in terms of cone responses from LGN to higher brain areas^9^ and a shift of selectivity toward intermediate hues in the MB diagram with more diverse selectivity in later processing stages^10^. Specifically, some suggested that hue-selective neurons in V1 have single-opponent receptive fields similar to LGN cells^11,12^ with comparable chromatic selectivities obtained by a rectified sum of the three cone types^13^ or by combining LGN activations in a nonlinear fashion^6^. In contrast, Wachtler *et al*.^14^ found that the tunings of V1 neurons are affected by context and different from LGN.

Although Namima *et al*.^15^ found neurons in V4, AIT and PIT to be luminance-dependent, others reported that in the Macaque extrastriate cortex, millimetre-sized neuron modules called globs, have luminance-invariant colour tunings^16^. Within globs in V4, clusters of hue-selective patches with sequential representation following the colour order in the hue, saturation and lightness (HSL) space were identified, which were called “rainbows of patches”^17^. Conway and Tsao^18^ suggested that cells in a glob are clustered by colour preference and form the hypothesized colour columns of Barlow^19^. Patches in each cluster have the same visual field location and overlap greatly in their visual field with their neighbouring patches^16,17^. Moreover, each colour activates 1–4 overlapping patches and neighbouring patches are activated for similar hues. Comparable findings in V2 were reported in^20^. Consequently, Li *et al*.^17^ suggested that different multi-patch patterns represent different hues, and such a combinatorial colour representation could encode the large space of physical colours, given the limited number of neurons in each cortical colour map. Other studies also suggested that glob populations uniformly represent colour space^21^ with narrow tunings for glob cells^21–23^. Similar findings for IT cells were reported by Zaidi *et al*.^24^.

Not only is there disagreement about the colour processing mechanisms in the visual cortex, but also which region in the brain represents unique hues. Furthermore, transformation mechanisms from cone-opponent responses to unique hues are unclear. While unique red is found to be close to the +L axis in the MB diagram, unique green, yellow and blue hues cluster around intermediate directions^25^, not along cone-opponent axes. Perhaps clustering of the majority of unique hues along intermediate directions could describe the suggestion by Wuerger *et al*.^26^ who proposed that the encoding of unique hues, unlike the tuning of LGN neurons, needs higher order mechanisms such as a piecewise linear model in terms of cone inputs. The possibility of unique hue representations in V1 and V2 was rejected in^27^, but like others^23,28^, they observed neurons in PIT show selectivities to all hue angles on the colour wheel and that there are more neurons selective to those close to unique hues. These findings were challenged in^29^. Similarly, Zaidi *et al*.^30^ observed no significance of unique hues in human subjects and responses of IT neurons, also confirmed in more recent studies^31,32^.

Among all the attempts to understand the neural processes for transformation from cone-opponent to perceptual colours, a number of computational models suggested mechanisms for this problem and other aspects of colour representation in higher areas^33–37^. These models, however, are one-layer formulations of perceptual hue encoding, or simply put, the totality of processing in these models is compressed into a single layer process. The end result may indeed provide a suitable model in the sense of its input-output characterization. However, it does not make an explicit statement about what each of the processing areas of the visual cortex are contributing to the overall result and they do not shed light upon the mystery of colour representation mechanisms in the brain.

In this article, we introduce a computational colour processing model that as Brown^38^ argues, helps in “understand[ing] how the elements of the brain work together to form functional units and ultimately generate the complex cognitive behaviours we study”. Accordingly, we build a hierarchical framework, inspired by neural mechanisms in the visual system, that explicitly models neurons in each of LGN, V1, V2, and V4 areas and reveals how each visual cortical area participates in the process. In this model, nonlinearity is gradually increased in the hierarchy as observed by^9^. Specifically, while a half-wave rectifier unit keeps the V1 tunings similar to those of LGN^13^, it makes V1 neurons nonlinear in terms of cone inputs. In V2, besides single-opponent cells, we propose employing neurons with multiplicative modulations^39–41^, which not only increase nonlinearity but also allow neuronal interactions by mixing the colour channels and narrows the tunings. De Valois *et al*.^36^ suggested that additive or subtractive modulation of cone-opponent cells with S-opponent cell responses rotates the cone-opponent axes to red-green and blue-yellow directions. We achieved this rotation with multiplicative modulations of L- and M-opponent cell activations with S-opponent neuron responses. We call these cells “multiplicative V2” neurons. Finally, V4 responses are computed by linearly combining V2 activations with weights determined according to tuning peak distances of V2 cells to the desired V4 neuron tuning peak.

**Figure 1:**
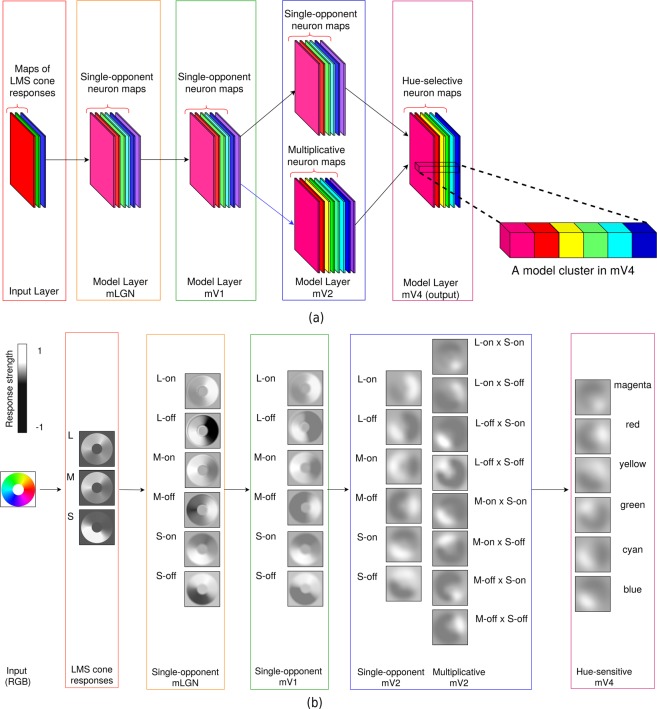
The proposed hierarchical model. (**a**) Our model architecture for local hue representation (best seen in colour). Each layer of this model implements neurons in a single brain area. The prefix “m” in each layer name represents model layers. Each map within a layer consists of neurons of a single type with receptive fields spanning the visual field of the model. The leftmost layer shows the input to the network with the LMS cone activations. The colour employed for each feature map in the model is figurative and not a true representation of the hue-selectivity of its comprising neurons. In layer mV4, a larger view of an example model cluster is shown, similar to the clusters found in monkey V4^17^. Each model cluster corresponds to a column of the three-dimensional matrix obtained by stacking mV4 maps. (**b**) An example of our model responses. We show each layer of the network within a rectangle, with a number of feature maps inside the rectangle. The selectivity of neurons is written next to each map. The receptive field of each neuron in these maps is centred at the corresponding pixel location. The neuron responses are shown in greyscale, with a minimum response as black, and maximum activation as white. The range of responses shown on the upper left part of (**b**) represents the minimum and maximum firing rate of model neurons. The dark border around each feature map is shown only for the purpose of this figure and is not part of the activity map.

Figure 1(a) depicts our proposed model and Fig. 1(b) demonstrates our network in action. Each layer of this model implements neurons in a single brain area. Each map within a layer consists of neurons of a single type with receptive fields spanning the visual field of the model; for example, a map of neurons selective to red hue in model layer V4. The leftmost layer in this figure shows the input to the network with the LMS cone activations. We found that the tuning peak of multiplicatively modulated V2 cells shifts toward hues along intermediate directions in the MB space. Consequently, these neurons have substantial input weights compared to single-opponent V2 cells to V4 neurons selective to hues along intermediate directions. Moreover, we observed a gradual decrease in distance of tuning peaks to unique hue angles reported by^42^ from our model LGN cells to V4 neurons. Our simulation results demonstrate that responses of our network neurons resemble those of biological colour cells.

In what follows, we will make a distinction between our model and brain areas by referring to those as layers and areas, respectively. That is, a set of model neurons implementing cells in a brain area will be referred to as a model layer. For example, model layer V2 implements cells in brain area V2. Moreover, whenever a brain area name is preceded with “m”, it is referring to the corresponding model layer, for example, mV2 for model layer V2. Additionally, although our model architecture looks similar to a convolutional neural network^43^, we emphasize that we employed no learning steps to determine the weights in our network, but rather set those according to existing biological evidence. When certain aspects of colour processing mechanisms in the brain are understood, there is no need to learn them. As such, we believe the significance of this study is its promising results with important implications for the *unknown* aspects of hue processing in the brain that were obtained without any data fitting. The rationale behind our choice is further discussed in Methods.

## Results

We designed simulation experiments to make two important aspects of our model clear:

1. at the single-cell level, our model neurons perform similarly to their biological counterparts.
2. at the system level, our hierarchical model, due to a gradual increase in nonlinearity, makes it possible to model neurons with narrow bandwidths, represent hues in intermediate directions, and represent unique hues in our output layer mV2.

As a result, our experiments bridge a mixture of single-cell examinations to evaluations of the hierarchical model as a whole.

### Model neuron tunings

**Figure 2:**
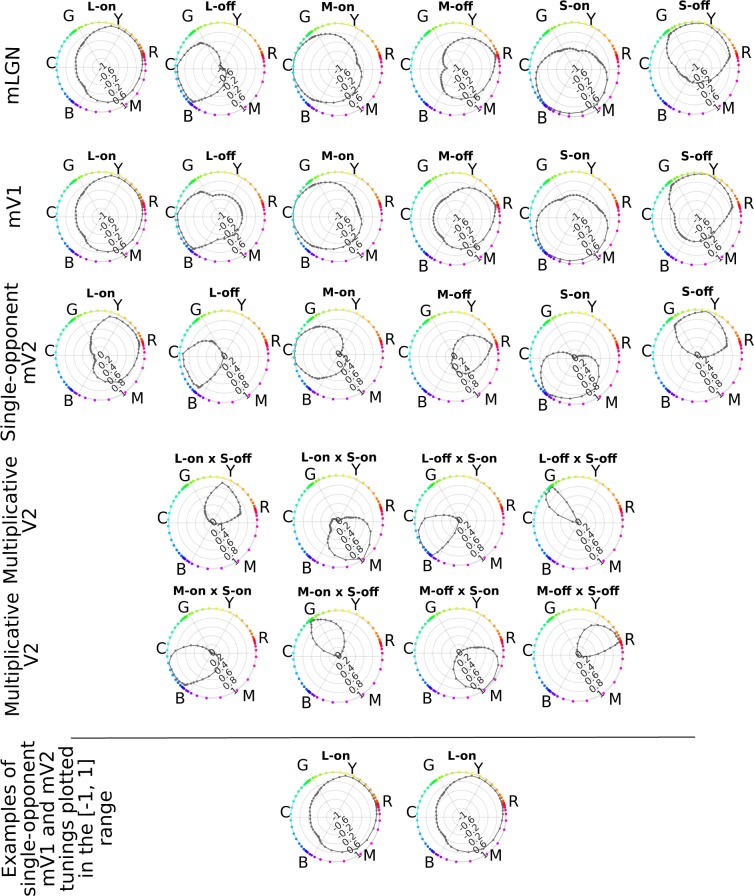
Model neuron tunings. Responses of neurons in layers mLGN, mV1, and mV2 to 60 hues sampled from the hue dimension in the HSL space. Each sampled hue is mapped to its corresponding hue angle in the MB space and is shown by a coloured dot corresponding to the sampled hue on the circumference of a unit circle in the MB space. Note that the positive vertical direction in the tuning plots corresponds to lime hues, following the plots from Conway and Tsao^18^, their Fig. 1. In each plot, the circular dimension represents the hue angle in the MB space. The level of responses is shown in the radial dimension in these plots with high activations away from the plot center. In each row, the model layer to which the neurons belong is specified on the left edge of the row. The neuron type is mentioned above each plot. In the bottom panel, we compare tunings of single-opponent mV1 and mV2 L-on neurons and show almost identical curves for these cells when plotted in the same range of [−1, 1].

To test the effectiveness of our approach in hue encoding, we examined the tuning of each hue-selective neuron in all layers of our network. We sampled the hue dimension of the HSL space, with saturation and lightness values constant and set to 1 and 0.5, respectively, following^17^. We sampled 60 different hues in the range of [0, 360) degrees, separated by 6 degrees. When these HSL hue angles are mapped to a unit circle in the MB space, they are not uniformly spaced on the circle and are rotated (depicted in Fig. 2). For example, the red hue in the HSL space at 0 deg corresponds to the hue at about 18 deg in the MB space.

**Figure 3:**
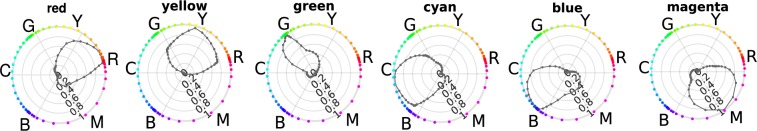
Model mV4 neuron responses to hues sampled from the hue dimension in the HSL space. The sampled hues are 6 degrees apart. In each plot, the angular dimension shows the hue angles in the MacLeod and Boyton diagram, and the radial dimension represents the response level of the neuron where larger responses are further away from the center. The dots on the circumference of each circle are representative of hues at sampled angles. The selectivity of each neuron is specified above its plot.

We presented all sampled hues to the model and recorded the activities of model neurons with responses shown in Figs. 2 and 3. Our single-opponent cells implement two types of center-surround receptive fields: X-on and X-off cells, where X represents one of the three cone types which dominates the input to the receptive field center and on/off signifies the excitatory/inhibitory contribution of that cone. The receptive field profiles of these neurons are borrowed from the studies by Wool *et al*.^2,3^.

In each plot, the circular dimension represents the hue angle in the MB diagram, and the radial dimension represents the neuron’s response level. We found mLGN and mV1 tunings look relatively similar with differences due to the nonlinear rectifier imposed on mV1 responses. We plotted the responses of mV1 neurons in both negative and positive ranges for comparison purposes with those of mLGN. For mV2 and mV4, the responses are shown in the positive range. Although it might not be evident from tunings of mV1 cells and single-opponent mV2 neurons in Fig. 2, due to the plotted range of responses in these figures, we emphasize that the tunings of these cells look identical when plotted in the same range, as the two examples in the bottom panel of Fig. 2 show. The average difference of responses between pairs of mV1 and their corresponding single-opponent mV2 cells is on the order of 10^−6^.

Comparing single-opponent and multiplicative mV2 tunings gives a clear image of narrower tunings in the S-modulated mV2 neurons. Not only do these cells have narrower tunings, but they generally peak close to intermediate directions. For instance, the mV2 L-off × S-off cell has a narrow tuning that peaks close to the unique green hue angle.

In mV4, we implemented six different neuron types according to distinct red, yellow, green, cyan, blue and magenta, which are 60 deg apart on the HSL hue circle. The weights from mV2 cells to mV4 neurons were determined according to the distance between peak activations of mV2 neurons to the desired hue in a mV4 cell. Tunings of mV4 neurons, depicted in Fig. 3, show a distinct peak for each cell close to its desired selectivity, with narrower tunings compared to single-opponent mV2 cells.

### Tuning bandwidths

**Figure 4:**
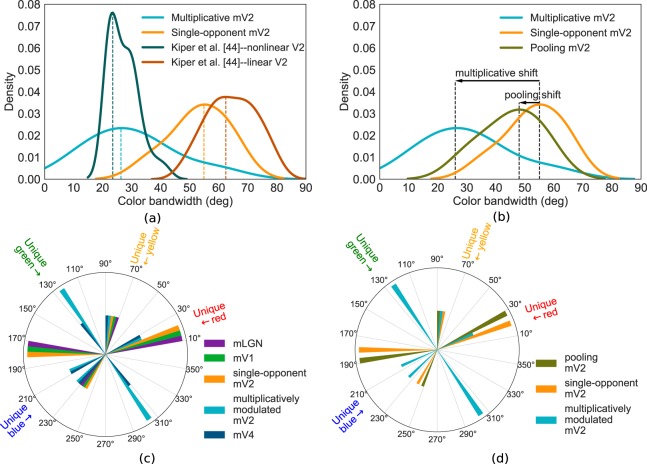
Tuning bandwidth and tuning peak analysis in the hierarchy. (**a**) Distributions of tuning bandwidths for mV2 neurons. Also, the bandwidth distributions of biological V2 cells reported by Kiper *et al*.^44^ are plotted for comparison. The distribution of multiplicative mV2 neurons is clearly separate and shifted toward smaller bandwidths compared to single-opponent mV2 cells, similar to the separate distribution of the V2 population reported by Kiper *et al*.^44^. This confirms our observation that multiplicative modulations result in narrower tunings. Dashed vertical lines indicate the bandwidth at which each distribution peaks. While multiplicative mV2 and biological nonlinear V2 distributions peak at around similar bandwidths, our single-opponent mV2 distribution is slightly shifted compared to that of biological linear V2 neurons. (**b**) Distributions of tuning bandwidths of single-opponent, multiplicative and pooling mV2 neurons. Although pooling decreases the tuning bandwidths, the bandwidth distributions of these neurons has a large overlap with that of single-opponent mV2 cells. Multiplicative mV2 cells, in contrast, shift the distribution to smaller bandwidths, separating it from that of single-opponent mV2 cells. The shifts in distributions of multiplicative and pooling mV2 cells with respect to single-opponent mV2 neurons are shown by annotated black arrows. (**c**) A polar histogram of selectivity peaks for our model neurons. Our mLGN, mV1 and single-opponent mV2 neurons cluster around cone-opponent axis directions while our model multiplicative mV2 and mV4 cells have peaks both close to cone-opponent and off cone-opponent hue directions. Note specifically that all model layers have neurons in polar bins containing unique red and unique yellow hue angles. Bins including unique green and unique blue angles are limited to multiplicative mV2 and mV4 types. (**d**) Peak selectivities of single-opponent, multiplicative, and pooling mV2 cells shown in a polar histogram of selectivities. Despite decreasing tuning bandwidths, pooling mechanisms do not shift the selectivities to intermediate directions, whereas multiplicative modulations both decrease the tuning bandwidths and shift selectivities to intermediate hue directions.

Kiper *et al*.^44^ defined tuning bandwidth as “the angular difference between the color vector giving the peak response, and that vector where the response had fallen to 50% of the difference between peak vector and 90 deg away”. They reported tuning bandwidth as a metric for tuning narrowness and observed separate bandwidth distributions of linear and nonlinear V2 cells. To obtain a quantitative evaluation of our observation with regards to narrower tunings due to multiplicative modulations, we computed the tuning bandwidth of mV2 neurons, following Kiper *et al*.^44^. For neurons exhibiting maximum activation at a range of neighboring hues (see mV2 M-on tuning in Fig. 2 as an example), we take the mean hue as the representative hue with peak response for computation of bandwidth and later for tuning peak. While our goal is to verify whether multiplicative modulations narrow the tunings, we did not hypothesize a model as Kiper *et al*.^44^ who computed the bandwidth threshold analytically for linear and nonlinear tunings, nor did we have a population of neurons to report the percentage of linear/nonlinear cells. Instead, we simply computed the bandwidth of responses for each cell type in the mV2 layer and plotted the distribution of tuning bandwidths. Specifically, we computed the tuning bandwidth of 6 single-opponent and 8 multiplicatively modulated mV2 neurons in our model. The bandwidth distributions are depicted in Fig. 4(a), where we also plotted the distributions of linear/nonlinear biological V2 cell population extracted from Kiper *et al*.^44^, their Fig. 3. Interestingly, bandwidth distributions of mV2 neurons are separate and overlap with that of biological linear and nonlinear V2 cells reported by Kiper *et al*.^44^. Although the distribution of single-opponent mV2 neurons is slightly shifted toward smaller bandwidths compared to that of biological linear V2 cells, both distributions are spread over a wide range of bandwidths. While the biological nonlinear V2 distribution is squeezed compared to that of multiplicative mV2 neurons, both distributions peak at similar bandwidths. Note that the area under all curves are normalized, hence, the higher peak in the nonlinear distribution. Single-opponent mV2 neurons are mainly clustered around large bandwidths, between 36 and 66 deg (mean = 52.98 deg). Tuning bandwidths of multiplicative mV2 cells vary between 9 and 60 deg with an apparent density toward bandwidths smaller than 40 deg (mean = 30.17 deg). These results confirm our previous observation that multiplicative modulations lead to narrower tunings.

Another approach to construct neurons with narrow tunings is to pool the responses of cells with similar selectivities in local neighbourhoods^45,46^. Koch *et al*.^46^ have shown this approach to narrow the tunings in orientation-selective cells. While multiplications cause a drop of firing rates and require an amplification of responses, the pooling approach does not reduce the firing rates. In order to compare the two methods and evaluate the effectiveness of each on narrowing the tunings, we implemented pooling in local regions of our mV2 layer, arguably the globs in V2. Our implementation follows the formulation of Koch *et al*.^46^, but for colour neurons. As the bandwidth distributions of mV2 neurons in Fig. 4(b) shows, we found that even though pooling narrows the tunings, multiplication has a more significant effect in decreasing the bandwidths. In other words, the shift toward smaller bandwidths with respect to the bandwidth distribution of single-opponent mV2 cells is larger for the bandwidth distribution of multiplicative cells compared to that of pooling mV2 cells, as shown by the two arrows in Fig. 4(b). We stress here that our results of narrower tunings of multiplicative mechanisms versus pooling does not indicate that the hue selective neurons do not employ pooling mechanisms. However, our model confirms that a simple feedforward S-modulated multiplication has more success in decreasing the bandwidth compared to pooling in globs.

### Tuning peaks

Watcher *et al*.^14^ observed that most neurons in V1 peak around non-opponent directions, while Kiper *et al*.^44^ reported that cells in V2 exhibited no preference to any particular colour direction and no obvious bias to unique hues. We tested the tuning peaks of model neurons to examine for cone-opponent vs. intermediate selectivities. Figure 4(c) shows a polar histogram of tuning peaks of all types of neurons in all layers of our model, where each sector of the circle represents a bin of the polar histogram. This figure clearly demonstrates that the majority of mLGN, mV1, and single-opponent mV2 cells (5 out of 6 neuron types) peak close to cone-opponent axes. In contrast, the family of multiplicative mV2 cells and hue-sensitive mV4 neurons peak at both cone-opponent and intermediate hues, as reported in^44^ and^21^. Simply put, with the multiplication mechanism, the representation of hues along intermediate directions starts to develop. This is in fact another advantage of multiplication over pooling of responses; multiplying the responses of cells with dissimilar selectivities rotates the peak responses to intermediate directions. In pooling, however, no shifting in the peak selectivities is achieved, as depicted in Fig. 4(d).

### Unique hue representation

**Figure 5:**
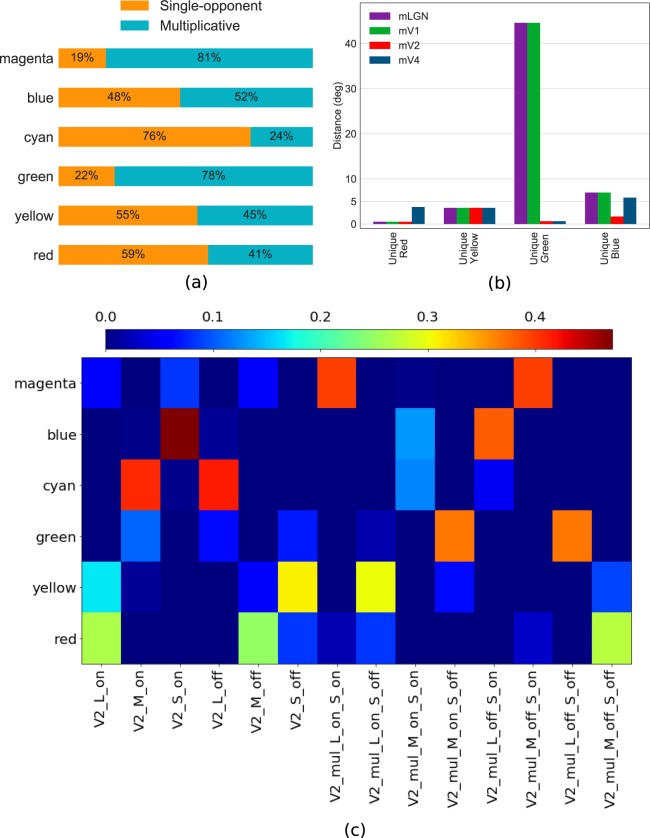
Multiplicative neurons contribute greatly in intermediate and unique hue representations. (**a**) Relative contributions of single-opponent and multiplicative mV2 cells to each hue-sensitive mV4 neuron. The orange and blue bars show the percentage of contribution from single-opponent and multiplicative mV2 cells to each specific mV4 cell, respectively. Multiplicative mV2 neurons have more substantial input weight to mV4 cells with selectivity in intermediate hue directions. Single-opponent mV2 cells make up most of the input weight to mV4 cells with peaks close to cone-opponent axes. (**b**) Distances of our model neurons, layer-by-layer, to unique hue angles reported by Miyahara^42^. Unique hue representation develops in the hierarchy. Note the significant drop in the peak distance for mV2 and mV4 neurons to unique green, achieved by increasing the nonlinearity in those layers. (**c**) The relative contributions of individual mV2 cells in the encoding of six distinct hues in our mV4 layer (best seen in colour). Each row represents a hue-sensitive mV4 cell, and each column shows a mV2 cell. Multiplicative mV2 cells are indicated as V2_mul_x_y, where x and y signify the type of mV1 neurons sending feedforward signal to the multiplicative mV2 cell. The weights are normalized to sum to 1.0. Determining weights from mV2 to mV4 layers in our network is described in detail in the Methods section.

In Fig. 4(c), each mV4 bar in the polar histogram is paired with a multiplicative mV2 bar. Wondering about the contribution of multiplicative mV2 cells to the responses of mV4 neurons, we compared the sum of single-opponent mV2 cell weights against that of multiplicative mV2 neurons, depicted in Fig. 5(a). Interestingly, mV4 cells selective to magenta, blue, and green hues, which are off the cone-opponent directions, have significant contributions from multiplicative mV2 cells. In green and magenta, in particular, multiplicative cells make up more than 78% of mV2-mV4 weights. The rest of hues, which are close to the cone-opponent directions in the MB space, receive a relatively large feedforward input from the single-opponent mV2 cells. In short, multiplicative cells play a significant role in the representation of hues in intermediate directions, while single-opponent cells have more substantial contributions to hues along cone-opponent axes.

Next, we asked the question of how well neurons in each of our model layers represent unique hues? To answer this, we computed the distance of peak angles for our model neurons to the unique hue angles reported by Miyahara^42^. For each unique hue, we report the minimum distance of peak angle among all neuron types in each layer to the given unique hue angle. As Fig. 5(b) shows, the distances to unique red and yellow are relatively small in all the four model layers, at less than 5 deg. This could be due to the fact that unique red and yellow reported in^42^ are close to cone-opponent axes in the MB space, in agreement with findings of^25^ for unique red. For unique green, there is a 44 deg drop in mV2 and mV4 distances compared to that of mV1. Also, the distance to unique blue further decreases in mV2, although with an increase in mV4. The increase in mV4 to unique blue and unique red, however, does not indicate that mV4 neurons cannot represent these unique hues as well as mV2 cells. Additional mV4 neurons with significant inputs from mV2 cells representing these unique hues can have a distance similar to mV2 cells. To summarize, we observed a gradual development in the exhibition of selectivity to unique green and blue hues, while selectivity to unique red and yellow was observed in early as well as higher layers. Moreover, these results suggest that mV2 cells with peak selectivities at less than 5 deg distance to unique hue angles, and consequently, neurons in higher layers, have the capacity to encode unique hues. Our results do not suggest any significance of unique hues, as was also reported in previous research^30–32^. We emphasize that our model neurons were not tuned to achieve unique hue representations. Instead, the results of this analysis, put together with that of Fig. 4(c), indicate that by introducing multiplicative nonlinearities, neurons with selectivities spanning the colour space start to emerge, some of which could potentially represent hues deemed as unique hues.

### Hue distance correlation

Li *et al*.^17^ found a correlation between pairs of stimulus hue distances and the cortical distances of maximally activated patches in each cluster. For this analysis, Li *et al*.^17^ employed an ordered representation for hues and defined the hue distances accordingly.

**Figure 6:**
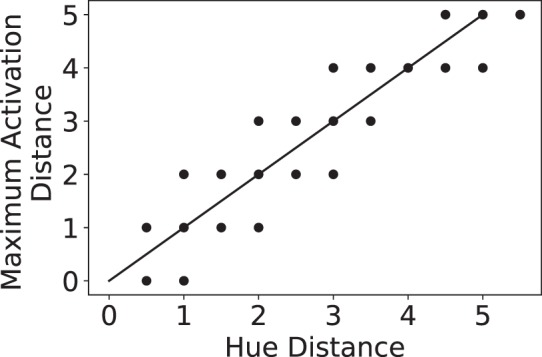
Hue distance correlation analysis between the hue distances and model patch distances in each model cluster. Our hierarchical model demonstrates a clear correlation between hue distances and the pattern of activations in mV4 neurons.

To test for a similar relationship between hue distances and the pattern of activities of mV4 neurons, we stacked our mV4 maps, in the order shown in Fig. 1(a), resulting in a three-dimensional array (details of this experiment are explained in Methods). We call each column of our stacked maps a model cluster and each element of the column a model patch. An example of is shown in Fig. 1(a). Similar to Li *et al*.^17^, we computed the distance of the two maximally activated model patches for a pair of hues. The plot in Fig. 6 demonstrates our model patch distances as a function of hue distances with a clear correlation (*r* = 0.93, *p* = 3.92 × 10^−30^). Simply put, similar to the biological V4 cells, the pattern of responses in our mV4 neurons is highly correlated with the ordering of hues in the HSL space.

### Hue reconstruction

Li *et al*.^17^ observed that 1–4 patches are needed to represent any hue in the visual field and that different hues were encoded with different multi-patch patterns. Then, they suggested that a combination of these activated patches can form a representation for the much larger space of physical colours.

Along this line, we show, through a few examples, that for a given hue, a linear combination of mV4 neurons can be learned and used for representing that particular hue. Although it would be impossible to learn weights for the infinitely many possible physical hues, we show a few examples as an instance of the possible mechanism for colour representation suggested by Li *et al*.^17^.

In this experiment, for a given hue value, we uniformly and independently sampled the saturation and lightness dimensions at 500 points resulting in 500 colours of the same hue. The goal is to compute a linear combination of mV4 neurons, which can reconstruct the groundtruth hue. The hues in this experiment were represented as a number in the (0, 2*π*] range. We performed an L1-regularized least square minimization^47^.

Table 1 shows some of the results for this experiment. Interestingly, in all cases, no more than four neuron types have large weights compared to the rest of the neurons, in agreement with findings of^17^. Specifically, in the case of red and yellow hues, about 99% of the contribution is from only a single cell, red and yellow neurons respectively. The last row in Table 1 is most insightful. It presents the weights for a lavender hue in equal distance from blue (240 deg) and magenta (300 deg). The weights for this example seem counter-intuitive as they include cyan and magenta with positive contributions, and blue is absent. However, careful scrutiny of peak angles for mV4 hues reveals that lavender hue at 270 deg is somewhere between the peaks for mV4 cyan (at 193 deg) and magenta (at 300 deg), and closer to magenta. This hue is mainly reconstructed from magenta, with more than 63% contribution, while the absence of blue is compensated with cyan, shifting the reconstruction from magenta toward lavender.

**Table 1:** The choice of weights for mV4 cells used for hue reconstruction in a few example hues.

Groundtruth hue (deg)	mV4 neuron					
	Red	Yellow	Green	Cyan	Blue	Magenta
red (360)	0.9934	0.0034	0.0006	0.0007	0.0006	0.0014
Yellow (60)	0.0010	0.9960	0.0008	0.0010	0.0004	0.0007
lavender (270)	0.0008	0.0019	0.0674	0.2946	0.0003	0.6351

Again, it must be stressed that this experiment was performed to examine the possibility of combinatorial representation mechanisms and a thorough investigation of this mechanism in the computational sense is left for future work. The examples shown here attest to the fact that intermediary hues encoded by mV4 neurons can indeed span the massive space of physical hues and are enough for reconstructing any arbitrary hue from this space.

## Discussion

Our goal was to further understanding of the colour processing mechanisms in the brain and to begin to assign colour representational roles to specific brain areas. We investigated the contributions of each visual area LGN, V1, V2, and V4 in hue representation by proposing a mechanistic computational model inspired by neural mechanisms in the visual system. Through a gradual increase in nonlinearity in terms of cone inputs, we observed a steady decrease in tuning bandwidths with a gradual shift in peak selectivities toward intermediate hue directions. Although one might be able to obtain the end result with a mathematical model in a single-layer fashion, such models do not lend insight to the neuronal mechanisms of colour processing in the brain. In contrast, not only do our model neurons in each individual layer exhibit behaviour similar to those of biological cells, but also at the system level, our hierarchical model as a whole provides a plausible process for the progression of hue representation in the brain. The main difference in terms of potential insight provided by a single-layer mathematical model and our work is that our model can make predictions about real neurons that can be tested. A model whose contributions are of the input-output behaviour kind cannot make predictions about component processes between input and output (see also^38^).

Multiplicative modulations in our model V2 layer increased nonlinearity in the hierarchy, narrowed tunings, and shifted tuning peaks toward intermediate directions. Our work suggests that the brain’s neurons also utilize such multiplicative mechanisms, which result in nonlinearity in their tunings and shifting of tuning peaks to intermediate directions, consistent with the report from Kiper *et al*.^44^. Such nonlinearities result in representations that span the colour space instead of reducing it to only “skeletons” of cardinal axes^24,45^. They also enable discrimination of slightly different hues, such as red and orange, by increasing the representational distances due to narrow tunings.

Our experimental results demonstrated that hue selectivity for mV4 neurons similar to that of neurons in area V4 of the monkey visual system was successfully achieved. Additionally, our observations from the hue reconstruction experiment confirmed the possibility of reconstructing the whole hue space using a combination of the hue-selective neurons in the mV4 layer. How this is achieved in the brain, for the infinitely many possible hues, remains to be investigated.

Finally, our hierarchical model provides an important suggestion about unique hue representations. Specifically, our computational experiments showed that as the visual signal moves through the hierarchy, responses with peaks close to unique hues start to develop; for example, for unique red as early as mLGN and for unique green delayed to mV2. Putting these together, we believe the answer to the question “which region in the brain represents unique hues?” is not limited to a single brain area, which in turn could be the source of disagreement among colour vision researchers. Instead, our findings suggest that this question must be asked for each individual unique hue. Then, putting answers for all unique hues together will result in a set of brain regions. In our model, {mLGN, mV2, mV4} is the answer. Although our model allowed us to make predictions about unique hues, our model neurons are not tuned for unique hues and the argument can be generalized for any arbitrary hue in intermediate directions.

In our model, adding a variety of neurons such as concentric and elongated double-opponent colour cells would result in a more comprehensive system. However, we did not intend to make predictions about all aspects of colour processing but only hue encoding mechanisms. We found that concentric double-opponent colour cells, for example, have tuning bandwidth distribution similar to single-opponent neurons and tuning peaks along cone-opponent axes. This finding suggests that the contributions of concentric double-opponent cells are comparable to those of single-opponent neurons for hue representation, but we did not investigate those contributions in other colour representations.

Our hierarchical model can be further extended to encode saturation and lightness. Also, we would like to address the problem of learning weights in the network for which current biological findings have no suggestion, for example, the set of weights from mV2 to mV4. Lastly, in order to keep our model simple and avoid second-order equations, we skipped lateral connections between neuron types. However, these are part of the future development of a second-order model for our network.

## Methods

**Figure 7:**
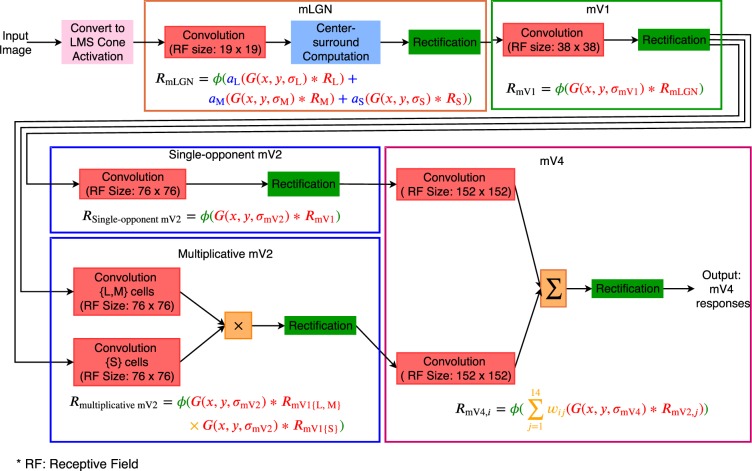
Hue processing network with details of computational blocks. Each layer is enclosed in a coloured rectangle similar to those shown in Fig. 1(a). Each little box in this figure represents one computational step in the model. The mathematical equation for each layer is included within the layer’s rectangle. The font colour of each component in the equation matches the colour of its corresponding computational box in the layer. For example, the font colour of the rectification function *ϕ* matches the green colour of the rectification box in mLGN, or the yellow colour for × in the multiplicative mV2 equation matches the yellow colour of the multiplication box in this layer. Note the arguments are delimited by the same-colour brackets. In convolution boxes, RF stands for receptive field.

In this work, the input to our model is LMS channels. In the event that the presented stimulus was available in RGB, we first performed a conversion into LMS channels using the transformation algorithm proposed by^48^ (we used the C code provided by the authors). As a result, one can think of the presented stimulus to the network as the activations of three cone types. These cone activations are fed to single-opponent mLGN cells, which in turn feed single-opponent mV1 cells with nonlinear rectification. In the mV2 layer, single-opponent neurons replicate the activations of those of mV1, but with larger receptive fields. Later, we refer to these single-opponent cells as “additive mV2 neurons”. “Multiplicative mV2” neurons form when single-opponent mV1 cells with L and M cone inputs are multiplicatively modulated by mV1 neurons with S-cone input. This approach is in a sense similar to S-modulations proposed by De Valois *et al*.^36^, but in a multiplicative manner and not additive or subtractive. Finally, the hue-sensitive neurons in mV4 receive feedforward signal from additive and multiplicative mV2 cells. Figure 7 depicts the computational steps of our model in each layer.

Our model was implemented in TarzaNN^49^. The neurons in all layers are linearly rectified. The rectification was performed using:

1 $$\phi (P)=(\begin{array}{cc}\tau , & {\rm{if}}\,mP+b < \tau \\ mP+b, & \,\,{\rm{if}}\,\tau \le mP+b\le s\\ \mathrm{1,} & {\rm{otherwise}},\end{array}$$

where *P* is neuron activity, and *m* and *b* are the slope and base spike rate respectively, *τ* is a lower threshold of activities and *s* represents the saturation threshold. This rectifier maps responses to [*τ*, 1]. Depending on the settings of parameters *τ* and *s*, and the range of activations for the model neurons, the rectifier might vary from being linear to nonlinear. Wherever this rectifier is employed in the rest of the paper, we mention the settings of the parameters, and whether parameter settings resulted in neuron activations to become linear or nonlinear in terms of their input.

The input to the hierarchical network was always resized to 256 × 256 pixels. The receptive field sizes, following^50^, double from one layer to the one above. Specifically, the receptive field sizes we employed were 19 × 19, 38 × 38, 76 × 76, and 152 × 152 pixels for mLGN, mV1, mV2, and mV4 layers respectively.

### Model LGN cells

The first layer of the hierarchy models single-opponent LGN cells. The mLGN cells are characterized by their opponent inputs from cones. For example, mLGN cells receiving excitatory input from L cones to their centres and inhibitory signals from L and M cones from the surround are known as L-on cells. Model mLGN cell responses were computed by^51^:

2 $$\begin{array}{rcl}{R}_{{\rm{mLGN}}} & = & \phi \,({a}_{{\rm{L}}}(G(x,y,{\sigma }_{{\rm{L}}})\ast {R}_{{\rm{L}}})+\,{a}_{{\rm{M}}}(G(x,y,{\sigma }_{{\rm{M}}})\ast {R}_{{\rm{M}}})\\  &  & +\,{a}_{{\rm{S}}}(G(x,y,{\sigma }_{{\rm{S}}})\ast {R}_{{\rm{S}}})-\,{a{\prime} }_{{\rm{L}}}(G(x,y,{\sigma {\prime} }_{{\rm{L}}})\ast {R}_{{\rm{L}}})\\  &  & -\,{a{\prime} }_{{\rm{M}}}(G(x,y,{\sigma {\prime} }_{{\rm{M}}})\ast {R}_{{\rm{M}}})-\,{a{\prime} }_{{\rm{S}}}(G(x,y,{\sigma {\prime} }_{{\rm{S}}})\ast {R}_{{\rm{S}}})),\end{array}$$

where * represents convolution. In this equation, mLGN response, *R*_mLGN_, is computed by first, linearly combining cone activities, *R*_L_, *R*_M_, and *R*_S_, convolved with normalized Gaussian kernels, *G*, of different standard deviations, *σ*, followed by a linear rectification, *ϕ*. For mLGN neurons, we set *τ* = −1 and *s* = 1 to ensure the responses of these neurons are linear combinations of the cone responses^6,13^. The differences in standard deviations of the Gaussian kernels ensure different spatial extents for each cone as described in^1^. Each weight in Eq. (2), determines presence/absence of the corresponding cone. The weights used for mLGN cells were set following the findings of^2^ and^3^. For example, for an L-on neuron, L-cones have excitatory contributions to the center and inhibitory surround contributions are from both L- and M- cones. In this case, for the center, *a*_L_ is positive with *a*_M_ and *a*_*S*_ set to zero, while in the surround only ${a{\prime} }_{S}$ is zero. In total, we modelled six different mLGN neuron types, L-on, L-off, M-on, M-off, S-on, and S-off. As an example, consider M-on cells. These neurons receive opposite contributions from L and M cones, while S cones with weight 0 exhibit no contribution. That is, M and L cones have excitatory and inhibitory effects respectively, while S cones are absent. This type of neuron is known to best respond to cyan-like hues^52^. A relatively similar hue selectivity is observed in L-off cells, which receive excitatory and inhibitory contributions from M and L cones respectively. Receiving such a pattern of input signal results in selectivities to cyan-like hues for both cell types. Despite this similarity, however, their responses indicate different messages. Specifically, strong responses of an M-on cell conveys high confidence in the existence of an M cone signal with less impact from L cone responses within its receptive field. In contrast, strong activations of an L-off cell indicates the existence of M cone signal with a confident message that almost no L cone activities exist within its receptive field. In other words, even a small amount of L cone responses within the receptive field of an L-off cell strongly suppresses the activation of this neuron. Looking at the feature maps for these two cell types in Fig. 1(b) reveals these slight differences in their selectivities. Note that while both neurons have relatively strong positive responses to the green and blue regions in the input, activations of the L-off cells, unlike the responses of M-on neurons, in the yellow region are strongly suppressed.

In what follows, whenever we refer to a cell as L, M, or S in layers mLGN and higher, we will be referring to the pair of on and off neurons in that layer. For instance, M-on and M-off neurons in mLGN might be called M neurons in this layer, for brevity.

### Model V1 cells

Local hue in V1, as suggested in^12^ and^11^, can be encoded by single-opponent cells. To obtain such a representation in the mV1 layer, the responses are determined by convolving input signals with a Gaussian kernel. Note that since single-opponency is implemented in the mLGN layer, by simply convolving mLGN signals with a Gaussian kernel, we will also have single-opponency in mV1. The local hue responses of mV1 were obtained by:

3 $${R}_{{\rm{mV1}}}=\phi (G(x,y,{\sigma }_{{\rm{mV1}}})\ast {R}_{{\rm{mLGN}}}),$$

where *ϕ* is the rectifier in Eq. (1). With *τ* = 0 and *s* = 1 for the rectifier, our mV1 neurons will be nonlinear functions of cone activations. In Eq. (3), substituting *R*_mLGN_ with any of the six mLGN neuron type responses will result in a corresponding mV1 neuron type. Therefore, there are six neuron types in layer mV1 corresponding to L-on, L-off, M-on, M-off, S-on, and S-off.

### Model V2 cells

In our network, the mV2 layer consists of two types of hue selective cells: single-opponent and multiplicative. The single-opponent neurons are obtained by:

4 $${R}_{{\rm{a}}{\rm{d}}{\rm{d}}{\rm{i}}{\rm{t}}{\rm{i}}{\rm{v}}{\rm{e}}{\rm{m}}{\rm{V}}2}=\phi (G(x,y,{\sigma }_{{\rm{m}}{\rm{V}}2})\ast {R}_{{\rm{m}}{\rm{V}}1}),$$

where *ϕ* is the rectifier in Eq. (1). With *τ* = 0 and *s* = 1 for the rectifier, the single-opponent mV2 cells are nonlinear functions of cone activations. In Eq. (4), substituting *R*_mVI_ with each of the six mV1 neuron type responses will yield a mV2 neuron type with similar selectivities, but with a larger receptive field. To be more specific, the responses of single-opponent mV2 neurons can be considered as a linear combination of mV1 activations.

To increase the nonlinearity as a function of cone activations in mV2, as observed by Hanazawa *et al*.^9^, and also to nudge the selectivities further toward intermediate hues, as found by Kuriki *et al*.^10^, we introduce multiplicative mV2 neurons. These cells not only add another form of nonlinearity to the model, other than that obtained by the rectifier in mV1, but also mix the different colour channels from mV1 and exhibit a decrease in their tuning bandwidths. In their model, De Valois *et al*.^36^ suggested that S-modulated neurons rotate the cone-opponent axes to perceptual-opponent directions. Their modulations with S activations were in the form of additions and subtractions, which does not add to the nonlinearity of neuron responses. We leverage their observation, but in the form of multiplicative modulations for additional nonlinearity. That is, each mV2 multiplicative cell response is the result of multiplying L or M neurons from mV1 with a mV1 S cell activations. For example, in Fig.1(b), “L-off × S-off” is for a cell obtained by modulating a mV1 L-off cell response by a mV1 S-off neuron activation. In our model, the multiplicative mV2 neurons are computed as:

5 $$\begin{array}{ccc}{R}_{{\rm{m}}{\rm{u}}{\rm{l}}{\rm{t}}{\rm{i}}{\rm{p}}{\rm{l}}{\rm{i}}{\rm{c}}{\rm{a}}{\rm{t}}{\rm{i}}{\rm{v}}{\rm{e}}{\rm{m}}{\rm{V}}2} & = & \phi (G(x,y,{\sigma }_{{\rm{m}}{\rm{V}}2})\ast {R}_{{\rm{m}}{\rm{V}}1\{{\rm{L}},{\rm{M}}\}}\times G(x,y,{\sigma }_{{\rm{m}}{\rm{V}}2})\ast {R}_{{\rm{m}}{\rm{V}}1\{{\rm{S}}\}}),\end{array}$$

where × represent multiplicative modulation, and ${R}_{{\rm{mV1}}\{{\rm{L}},{\rm{M}}\}}$ and ${R}_{{\rm{mV1}}\{{\rm{S}}\}}$ are for responses of an L or M cell and S neuron from mV1 respectively. As before, *ϕ* is the rectifier from Eq. (1) with the same parameters as those of the additive mV2 cells. Multiplicative mV2 cells are nonlinear with respect to cone inputs and bilinear with regards to mV1 activations.

Multiplicative mV2 neurons have narrower bandwidths than those of additive mV2 cells, which we showed quantitatively earlier. However, consider the multiplicative mV2 maps in Fig. 1(b) for a brief qualitative explanation. For the hue wheel as input, relatively high responses of the single-opponent mV2 cells span a larger region of their map compared to multiplicative mV2 cells. This is an indication that multiplicative mV2 cells are selective to a narrower range of hue angles. As an example, both L-off and S-off mV1 cells have high activations for relatively large regions of the input respectively. However, when multiplied, the resulting neuron, i.e. the “L-off × S-off” cell has strong responses for regions with greenish colour, and the activation of the L-off mV1 cell to bluish regions is suppressed to the extent that the L-off × S-off cell shows close to no responses.

As a summary, in layer mV2, a total of 14 neuron types are implemented: 6 additive and 8 multiplicative cell types.

### Model V4 cells

We modelled mV4 neurons representing local hue using a weighted sum of convolutions over mV2 neuron outputs. More specifically, responses of the *i*-th mV4 neuron, ${R}_{{\rm{mV4}},i}$, are computed as:

6 $${R}_{{\rm{mV4}},i}=\phi (\mathop{\sum }\limits_{j=1}^{14}\,{w}_{ij}(G(x,y,{\sigma }_{{\rm{mV4}}})\ast {R}_{{\rm{mV2}},j})),$$

where ${R}_{{\rm{mV2}},j}$ represents the responses of the *j*-th mV2 neuron, and *ϕ* is the rectifier introduced in Eq. (1), with *τ* = 0 and *s* = 1. As a result of this parameter setting for the rectifier, each mV4 cell is a linear combination of mV2 cell responses and hence, nonlinear in terms of cone inputs. The set of weights {*w*_*ij*_}_*j*=1, …, 14_ determine the hue to which the *i*-th model mV4 neuron shows selectivity.

In layer mV4, we implemented six different neuron types according to distinct hues: red, yellow, green, cyan, blue, and magenta. The chosen hues are 60 deg apart on the hue circle of HSL, with red at 0 deg. These hues were also employed in the V4 colour map study^17^ and for comparison purposes, we utilize these hues. When the six mV4 colours are mapped to the MB space, the hue angles are shifted with respect to those of HSL, with red, yellow and cyan hues close to cone-opponent axes in the MB space, and green, blue and magenta along intermediate directions. Although here we limit the number of modelled neuron types in this layer to six, we would like to emphasize that changes in combination weights will lead to neurons with various hue selectivities in this layer. Modelling neurons with selectivities to a wide variety of hues with yet narrower tunings could be accomplished in higher layers, such as IT, by combining hue-selective model neurons in mV4.

In order to determine the weights from mV2 to mV4 neurons, the *w*_*ij*_’s in Eq. (6), we considered the distance between peak activations of mV2 neurons to the desired hue in a mV4 cell. The hue angle between these two hues on the hue circle is represented by *d*_*ij*_. Then, the weight *w*_*ij*_ from mV2 neuron *j* to mV4 neuron *i* is determined by:

7 $${w}_{ij}=\frac{{\mathscr{N}}({d}_{ij};0,\sigma )}{{Z}_{i}},$$

where ${\mathscr{N}}(.;0,\sigma )$ represents a normal distribution with 0 mean and *σ* standard deviation, and *Z*_*i*_ is a normalizing constant obtained by

8 $${Z}_{i}=\mathop{\sum }\limits_{j=1}^{14}\,{\mathscr{N}}({d}_{ij};0,\sigma ).$$

The weights used for each of mV4 neuron types are summarized in Fig. 5(c). In this figure, each row represents the weights for a single mV4 cell, and the columns are for mV2 cells. Note that all mV2 to mV4 weights are normalized to sum to 1.0. That is, the sum of weights in each row is 1. In this figure, dark red shows a large contribution, while dark blue represents close to no input from the relevant mV2 neuron. Consider, for example, the weights for the red mV4 cells. This neuron has relatively large weights from mV2 L-on, M-off, and M-off × S-off cells. In other words, cells with large contributions from L cones. This observation is not surprising as previous research by Webster *et al*.^25^ found that unique red in human subjects has the largest contributions from L cones.

In Fig. 1(b), at the mV4 layer, from top to bottom, the neurons selective to magenta, red, yellow, green, cyan, and blue are displayed. As expected, mV4 yellow neurons, for instance, show activations across red, yellow, and green regions of the stimulus, with stronger activations in the yellow segment.

### Hue distance correlation

For the hue distance correlation analysis, Li *et al*.^17^ employed an ordered representation for hues, according to the sequence ordering of patches witnessed in clusters, with 0 for magenta, 1 for red, and so on. They defined the hue distances as the difference of these assigned values.

For a similar analysis, we stacked our mV4 maps, in the order shown in Fig. 1(a), beginning with magenta, red, and so on. Stacking these maps results in a three-dimensional array, each column of which can be interpreted as a cluster of hue-selective patches, with neighbouring patches sensitive to related hues, similar to those observed in V4^17^. We call each column of our stacked maps a model cluster and each element of the column a model patch. An example of a mV4 cluster in a larger view is shown in Fig. 1(a). For a given model cluster and a pair of stimulus hues, we compute the distance of the two maximally activated model patches. For example, the distance of the red patch from the cyan patch as shown in Fig. 1(a) is 4. In this experiment, we employed our sampled hues from the HSL space, starting from red at 0 deg, separated 30 degrees, resulting in 12 stimulus hues. Similar to the ordering assigned to stimulus hues employed in^17^, we assigned values in the range [0, 5.5] at 0.5 steps starting with 0 for magenta.

### Choice of the model

Looking back at our network architecture in Fig. 1(a), and also the computational operations for each layer of our model, one might notice similarities with a convolutional neural network (CNN)^43^ that learns the weights between model layers. After all, our network architecture is similar to that of a CNN: the responses of neurons in each layer of the model are computed by a convolution followed by a rectification, similar to the operations in a CNN. Despite these similarities, our model differs from a CNN as not only do we not learn the weights in our network, but also that we set the parameters in our model according to reported properties of neurons in each brain area that we modelled in our hue processing network. We emphasize here that our choice of the model differs from a CNN for the following reasons:

1. Our goal was to introduce a biologically inspired model that would help in understanding hue encoding mechanisms in the brain. In doing so, we designed each neuron in our network according to the existing findings of the brain. For example, the receptive field profile and the weights from cones to single-opponent cells in our model LGN layer were set based on the reported findings of Wool *et al*.^2,3^. In a CNN, these parameters of the model are learned from data, and as a result, any receptive field profile and any setting of weights might be learned, which could possibly be different from those of biological colour neurons. Similar to our discussion about one-layer models, in an end-to-end manner, CNNs might succeed in hue representation and specifically in the encoding of unique hues. However, the individual neurons in such models might not match with those of the brain and hence, will not demystify colour processing in the brain.
2. One challenge in convolutional neural networks is interpreting the learned features in the hidden layers. Often, the learned features in the first hidden layer compare well with biological V1 neurons. However, learned features in deeper layers are difficult to explain. There have been attempts to understand and interpret hidden layer features^53,54^. However, a clear understanding of learned features and their semantic interpretation is yet to be achieved. As a result, had we employed a CNN model, we may have been impeded in explaining the functional and representational roles of each layer of the model as was our goal.
3. In this work, we did not have access to any cell recording data. Nonetheless, even with such data accessible to us, we would not have been able to use a CNN model. Often, cell recording data is limited and sparse and not enough for learning the massive number of parameters in a CNN. As an example, consider LeNet5^43^ with 60 K trainable parameters from three convolutional layers, two subsampling layers, and one fully-connected layer. In our model, we have three convolutional layers and one fully-connected layer comprising 500 K free parameters. The architecture of LeNet5 is close to ours and makes good ground for comparison. Note that the difference in the number of parameters between LeNet5 and our network is due to the tiny receptive field sizes in LeNet5 compared to ours. For example, the first convolutional layer in LeNet5 has neurons with receptive field sizes 5 × 5 while our mLGN cells have receptive fields of size 19 × 19. In the handwritten recognition task that LeNet5 was tested on, the MNIST dataset consisted of 60 K training samples for 10 digit classes. The minimum number of samples per class in this dataset is 5,421. Requiring the same number of samples per class for training our model means that we will need 5,421 samples per hue class. We have six hue classes in our output layer mV4. Therefore, we will need at least 5,421 × 6 = 32,526 cell recordings with 5,421 recordings of neurons selective to each of the six different hues in our model. As another example, AlexNet^55^ with 5 convolutional layers and 3 fully connected layers, in addition to other layers such as dropout, has an architecture, among state-of-the-art CNN models, closest to that of our model. AlexNet with 60 million parameters was trained on the ILSVRC dataset^56^. The dataset in 2012, the year AlexNet was introduced, had a minimum of 732 images per class with a total of 1000 image classes. For training a CNN model such as AlexNet, 732 samples per class was not enough and as a result, a data augmentation step increased the number of samples to more than 2.9 million images per class and a total of more than 5.2 billion images in the dataset. Now, let us assume that 732 samples per class is enough. Therefore, the total number of recordings required to train the model with 6 hue classes in mV4 will be at least 732 × 6 = 4,392 samples. Although this value is less than the number of samples computed earlier following the MNIST example, it is still by far more than what is usually reported in physiological studies with the number of cell recordings of only a few hundreds. Note that often the actual number of samples in cell recordings is far less as many of the recorded cells are classified as non-hue-selective.

We acknowledge that a certain set of parameters in our model were set according to biological findings and the remaining parameters, such as the weights from our layer mV2 to mV4, were set heuristically. Indeed, a learning algorithm, in this case, might prove to be helpful in making predictions about these connections in the brain. This step, as described in the Discussion section, is left to be further explored in the future.

### Accession codes

Our code and data are available on the Open Science Framework (OSF) website at: https://osf.io/kjz47/?view_only=c6db9ea547294e53ba4ad5c2d8a8a4b6.

## Data Availability

Our code and data are available on the Open Science Framework (OSF) website at: https://osf.io/kjz47/?view_only=c6db9ea547294e53ba4ad5c2d8a8a4b6.
